# Brazilian Vegetarian Population—Influence of Type of Diet, Motivation and Sociodemographic Variables on Quality of Life Measured by Specific Tool (VEGQOL)

**DOI:** 10.3390/nu12051406

**Published:** 2020-05-14

**Authors:** Shila Minari Hargreaves, Eduardo Yoshio Nakano, Renata Puppin Zandonadi

**Affiliations:** 1 Department of Nutrition, Faculty of Health Sciences, University of Brasilia (UnB), Campus Darcy Ribeiro, Asa Norte, Brasilia DF 70910-900, Brazil; renatapz@yahoo.com.br; 2 Department of Statistics, University of Brasilia, Brasilia DF 70910-900, Brazil; eynakano@gmail.com

**Keywords:** vegetarian diet, vegetarianism, quality of life, questionnaire, motivation

## Abstract

The adoption of a vegetarian diet has been associated with positive health outcomes. However, few studies evaluate the effect of this eating pattern on quality of life. Moreover, no specific instrument for the vegetarian population to measure the quality of life is available worldwide. Therefore, this study aimed to elaborate and validate a specific questionnaire to measure the quality of life in vegetarians. The Specific Vegetarian Quality of Life Questionnaire (VEGQOL) was constructed based on other instruments and studies related to vegetarianism. The content and semantic validation were performed by a group of experts, followed by a pilot study to evaluate the questionnaire acceptability and reproducibility. Discriminant validation was tested using the WHOQOL as the gold standard measure (Pearson correlation ranging from 0.302 of the domain 3 to 0.392 of the domain 2). Afterward, a nationwide survey was conducted using VEGQOL. Content and semantic validation selected 19 of the initial 30 items. VEGQOL presented good reproducibility (Cohen’s Kappa coefficient ranging from 0.361 to 0.730 and intraclass correlation coefficient of 0.820) and internal consistency (0.708), both adequate to evaluate the quality of life in vegetarians. The sample size (*n* = 5014 individuals, error of 3% at a level of significance of 5%) and distribution was representative of the Brazilian vegetarian population. In general, the quality of life of Brazilian vegetarians was considered satisfactory (VEGQOL cut off points 70–80). Among different types of vegetarians, the vegans showed better results with a VEGQOL mean value of 79.2 ± 10.7. Older individuals, the ones who adopted the diet for a longer time (VEGQOL mean value of 75.8 ± 12.7) and the ones who had other vegetarians in their social network (VEGQOL mean value of 74.6 ± 12.2) also had a better quality of life score. Individuals who adopted it for ethical or health reasons had a higher quality of life score. The questionnaire produced in this study is a useful tool for future research in this area. Results were better for vegans and for the ones who adopt the diet for ethical or health reasons.

## 1. Introduction

Vegetarianism is defined as dietary patterns varying according to consumption restriction degree and it is influenced by cultural, religious, regional and individual factors [1,2]. Despite speculations that primitive men lived mainly on an almost vegetarian diet, the earlier official reports about vegetarianism date back to ancient Egypt and India adopted mostly for spiritual reasons [3,4,5]. Over the centuries, vegetarianism has been adopted for religious, philosophical, ethical, sustainability and health reasons in different societies worldwide [6,7]. However, it was only in the twentieth century that it started becoming a common practice, growing stronger as a movement, up to the present moment [8]. Especially over the last years, vegetarianism has gained more visibility and followers. From a global perspective, Asia is the continent with the highest prevalence, with 19% of the population being vegetarian. Africa and the Middle East’s prevalence of vegetarianism is 16%, followed by 8% in South and Central America and 6% in North America. Europe has the lowest prevalence, with 5% of vegetarians in the population [9]. From 2012 to 2018, the number of vegetarians in Brazil increased from 8% to 14%, representing a significant portion of the population [10].

According to the Brazilian Vegetarian Society, vegetarians are “those who exclude from their diet all types of meat, poultry and fish and their byproducts, with or without dairy products and eggs’ consumption” [11]. Vegetarian diets can be separated into four main categories—semi-vegetarian or flexitarian (consumption of meat limited to once per week or non-consumption of red meat); pesco-vegetarian (exclusion of all meats except fish and seafood); ovo-lacto-vegetarian (exclusion of all types of meats but not eggs and dairy products); and strict vegetarian or vegan (excludes any food of animal origin) [12,13].

Despite the progress related to the knowledge about vegetarianism, due to rapid growth of vegetarianism adoption, new research is necessary to evaluate its prevalence, as well as motivations related to the practice; psychological and behavioral influences; and the differences between population groups, gender and age of individuals [14]. To date, data on the effect of vegetarian diets are still sparse in the literature. Even in meta-analysis and review studies, lack of standardization creates barriers to result in analysis and many studies focus only on health parameters [15,16,17].

In addition to the health effect, it is known that the adoption of a food pattern can influence the quality of life (QoL) of the individual adopting it [18]. According to the World Health Organization (WHO), QoL is a multifactorial concept that includes the following dimensions—physical (physical state), psychological (affective and cognitive state), social (interpersonal relations and social roles in the life of individuals) and spiritual (“meaning of life” and personal beliefs). Therefore, QoL is defined as “individual perception of their position in life, in the context of the culture and value systems in which they live and in relation to their goals, expectations, patterns and fears” [19].

It is known that restrictive diets have an impact on the individuals’ QoL, potentially being both positively and negatively [18]. Because vegetarianism is a restrictive dietary pattern, it may result in negative impacts on QoL, such as those observed in other restrictive diets (difficulty in socializing, lack of practicality, exclusion feelings, difficulty finding food in the market, prices, among others) [20,21]. However, the adoption of a vegetarian diet, unlike other restrictive diets (e.g., in gluten-related disorders, milk-related disorders, glycose/sucrose-related disorders, etc.), is usually a result of a personal choice, not a necessity for treatment purposes. In this way, it is possible that the aspects associated with well-being, personal satisfaction and the idea of engagement in a larger cause (protection of animals and environment or spiritual benefits) could have a positive influence on the individuals’ QoL.

Some studies have been able to demonstrate that, in general, vegetarianism has a positive effect on the QoL of those who adopt it. A pilot study conducted in the United States with individuals who stayed at a raw vegan institute for one to three weeks, with a 12-week follow up (*n* = 51) showed improvements in mental and emotional QoL of participants at the end of the study. [22]. A quasi-experimental study conducted in the United States with 292 diabetic overweight participants showed that participants who followed a vegan diet for 18 weeks had an improvement in QoL parameters. [23]. A randomized open trial was also conducted with diabetic patients (*n* = 74), showing that a vegetarian diet led to greater improvements in QoL and mood when compared to a standard diet for type 2 diabetes [24]. The effect of a vegan diet on diabetics and/or overweight individuals was also tested on a corporate site, where employees were allocated to follow a vegan diet (*n* = 68) or received no diet instruction (*n* = 45) over 22 weeks. The intervention group had improvements in QoL parameters such as mental health, general health and overall satisfaction compared to the control group [25]. QoL was also measured in healthy individuals in a cross-sectional study conducted with 123 omnivores and 158 vegetarian and vegan runners in German. The study showed that all participants had good QoL regardless of the type of diet [26]. Despite a few evidence already reported showing potential benefits of a vegetarian diet on QoL, the data volume on this subject is still scarce and the lack of a specific instrument that evaluates the QoL focused on the vegetarian population makes standardization and evaluation harder.

Assessing the influence of vegetarianism on QoL in a global and standardized way is fundamental for obtaining more accurate data needed to better base health professionals’ conduct, public policies and even market reactions related to vegetarianism. Therefore, this study aimed to develop a specific instrument to measure QoL in vegetarians and to evaluate the influence of the vegetarian diet on the QoL of Brazilian vegetarian population.

## 2. Materials and Methods

### 2.1. Study Design

A cross-sectional research was carried out in four steps—(I) selection and organization of the instruments used to evaluate the QoL; (II) development of a specific Vegetarian Quality of Life Questionnaire; (III) internal validation (content and semantic validation, reproducibility and internal consistency) of the specific instrument; (IV) online application of the questionnaire for external validation.

### 2.2. Selection and Organization of the Study Instruments

The instrument was organized in three parts to evaluate the vegetarians’ QoL—(I) sociodemographic data, to characterize our study sample; questions of self-referred weight and high to calculate body mass index (BMI); (II) specific QoL questionnaire for vegetarians, developed and validated for this study, as described below; (III) general QoL questionnaire (WHOQOL-BREF [27]), selected based on literature research to evaluate aspects not directly related to the vegetarian diet and to be used as one of the validation tools.

### 2.3. Development of the Vegetarian Quality of Life Questionnaire (VEGQOL)

The Vegetarian Quality of Life Questionnaire (VEGQOL) was developed based on Quality of Life Associated with Dietary Change Questionnaire [28], which evaluates taste satisfaction, cost, convenience, meal planning and healthcare parameters. This questionnaire was chosen as a basis because of its focus on the impact of dietary changes on QoL. General QoL instruments, such as the WHOQOL-BREF have already been developed and validated to evaluate aspects such as general health, psychological status, environmental aspects, social relationships, spirituality and physical capacity [27]. Therefore, we focused on dietary aspects, as well as potential consequences of a new dietary pattern adoption on QoL, defining the key points of the specific QoL questionnaire for vegetarians. The selected instrument was translated with the method described by Bullinger et al. [29]. In this method, two translators who are English-fluent and Portuguese native speakers independently translate the questionnaire to Portuguese. Potential discrepancies between the two versions were solved with health experts’ help. The final Portuguese version was then retranslated to English by two bilingual individuals. At the end of the process, translators come together to reach a consensus.

The Portuguese version was then adapted to encompass all specific aspects related to the vegetarian diet. An extensive scientific literature review was carried out to gather material to compose the questionnaire. Data from studies conducted with the vegetarian population were used to include relevant questions related to the vegetarian diet. All gathered information was used to elaborate a preliminary version of the questionnaire, which was then submitted to content and semantic validation [30,31].

### 2.4. VEGQOL Validation

#### 2.4.1. Content and Semantic Validation

Content and semantic validation were carried out using the Delphi method [29]. Professors of the University of Brasília Dietitian course with expertise in questionnaire construction and validation, as well as vegetarian health professionals and individuals with at least a master’s degree level, were invited to participate as judges (*n* = 10). They were contacted by e-mail containing an invitation letter and consent form.

According to Delphi’s method [29], experts must evaluate the instrument content. In the first round, judges evaluate each question’s relevance, indicating if they agree or not with its maintenance in the questionnaire. A 5-point Likert scale was used for it, with the following possible answers—1—“I totally disagree with the item”; 2—“I partially disagree with the item”; 3—“I neither agree nor disagree with the item”; 4—“I partially agree with the item”; and 5—“I fully agree with the item.” For each item, judges also could write comments to suggest changes, as well as include or exclude items. In case of disagreement among judges, the questionnaire was adjusted and forwarded to a second round, until a consensus is reached [30].

The semantic evaluation was performed simultaneously with content evaluation. Judges were asked to evaluate each item regarding clarity and understanding. Likert scale was used as follows—0—“I did not understand it at all”; 1—“I understood it a little”; 2—“I somewhat understood it”; 3—“I understood almost everything but I had some questions”; 4—“I understood almost everything”; 5—“I understood it perfectly and had no questions.” Once again, in case of disagreement, a new round would be conducted after adjustments, until consensus was reached.

The mean grade for the evaluation of content and semantic evaluation of each item was calculated considering the judges’ responses. The degree of agreement among the experts for each item was evaluated through the Kendall (W, ranging from 0 to 1). W-values ≥ 0.66 indicates that the judges applied the same standards of evaluation. W-values < 0.66 suggest disagreement among the judges [32]. The criteria for the approval of the item were W-values ≥ 0.8, with the minimal agreement of 80% among judges [30].

#### 2.4.2. Pilot Study

After the questionnaire construction completion, a pilot study was conducted to evaluate its reproducibility, virtual application feasibility and internal consistency of the items. In this step, a minimum of 20 vegetarian individuals would be conveniently selected to fill the printed version of the questionnaire. Two to three days after completion, the same individuals were asked, without prior notice, to complete the same questionnaire in the virtual version (using the SurveyMonkey^®^ tool—San Matteo, CA, USA) to verify the reproducibility of the instrument by test-retest and validate the virtual application of the instrument. In addition, data obtained in this step was analyzed to verify the internal consistency of the items that make up the VEGQOL.

#### 2.4.3. Questionnaire Validation

Questionnaire validation was carried out by the following five steps—(a) validation of content and semantics by the technique of judges, previously described; (b) acceptability of the questionnaire, measured by the number of missing items. According to Fitzpatrick, Davey, Buxton, & Jones [33], it is essential that high response rates are obtained in self-administered questionnaires, to facilitate the interpretation and generalization of data and avoid biases due to lack of response. The number of missing items was calculated based on the total left blank items in the pilot study and considered acceptable if compared to other studies that used QoL questionnaires. For this, the virtual survey allowed questions to be skipped in the pilot study; (c) reproducibility of the test, measured by the test-retest, comparing the responses of each individual to printed and virtual questionnaire. An Intraclass Correlation Coefficient of 0.7 or higher was considered acceptable [34]; the results of this step were also used to validate the application of the questionnaire virtually; (d) internal consistency of items that compose the VEGQOL score, which were measured by Cronbach’s alpha method [35]. A reliability level of at least 0.7 is considered acceptable [36]; and (e) discriminant validation, by correlating the score results of the VEGQOL with each WHOQOL domain result (Considering WHOQOL as the gold standard measure to evaluate the QoL).

### 2.5. Questionnaire Application

After the validation step, the instrument application was carried out with SurveyMonkey^®^ tool. The weblink to access the research was sent via email, messaging apps and social networks. Volunteers were recruited nationwide with the help of vegetarianism support groups as well as media outreach to reach as many vegetarians as possible. Volunteers received, together with the research link, the invitation to participate, as well as the Consent Form.

Vegetarian adults (over 18 years of age) from the entire country were recruited to participate in the study so that the QoL of this population group in Brazil could be traced. Ethical approval was obtained for this study by the Ethics Committee University of Brasília’s Health Institute (protocol number: 94114118.7.0000.0030). The study was conducted according to the guidelines laid down in the Declaration of Helsinki and followed the Recommendations for the Conduct, Reporting, Editing and Publication of Scholarly work in Medical Journals.

No official data regarding vegetarian population distribution among Brazilian regions had been published. Therefore, the sampling size was calculated based on data from MapaVeg, a national project that conducts a poll to evaluate vegetarian population distribution in Brazil (*n* = 29,282). The sampling size was calculated as described by Hair et al. [37], considering an error (e) of 3% and a level of significance (α) of 5%. The minimum estimated sample size would be of 1030 participants.

### 2.6. Statistical Analysis

The categorical variable descriptions were presented as frequencies and percentages; the quantitative variables were described regarding mean and standard deviation. The confidence intervals of the percentages were calculated by normal approximation. The reproducibility of the test-retest responses was verified using Cohen’s kappa coefficient (for categorical variables) and by the intraclass correlation coefficient (ICC) (for quantitative variables). The internal consistency of the questionnaire was measured using Cronbach’s alpha method. The discriminant validity was measured by comparing the results from VEGQOL and WHOQOL-BREF domains through Pearson’s chi-square test. In the case of BMI, this comparison was performed using the Analysis of Variance (ANOVA) followed by Tukey *post-hoc* test. The Kolmogorov-Smirnov test verified normality. All analyses considered bilateral hypotheses and a significance level of 5% (*p* < 0.05). The analyses were performed by the IBM SPSS Statistics for Windows (Armonk, NY: IBM Corp) and Microsoft Excel. The study hypothesis was specified before the data were collected. The analytic plan was pre-specified and any data-driven analyses are clearly identified and discussed appropriately.

## 3. Results

### 3.1. Selection and Organization of the Study Instruments

The final questionnaire used in the study was composed of three parts, described below.

#### 3.1.1. Sociodemographic Data

The first part of the instrument included variables commonly used to describe populations. We used data from the Brazilian National Institute of Geography and Statistics (*Instituto Nacional de Geografia e Estatística–IBGE*) as a reference. Gender, age, income, education level and housing location were the included variables [38].

Moreover, referred weight and high were included in this part to calculate body mass index (BMI), used to classify the individuals’ nutritional status [39]. Despite being an indirect method, referred weight and high in the adult population has good compliance and validation, being considered a feasible tool in studies where it is not possible to do direct measure [40].

#### 3.1.2. Quality of Life Related to the Vegetarian Diet

The instrument proposed by Delahanty et al. [28] was adapted to our study (as described in Section 3.2). Firstly, it was translated into the Portuguese language and then adapted to compose the second part of the instrument of this research. In addition to the questions that assessed the QoL, others were included in this part of the instrument to help characterize the study population, namely—(a) type of adopted diet (sub-classifications of vegetarian diets). For this, although there is no consensus, a classification that most frequently appears in studies with this public was used as a reference, to allow future comparison with data from the scientific literature. Examples of studies using such a classification are the Health Adventist Study 2 [41] and the study by Clarys et al. [12]; (b) primary motivation for the adoption of the diet. In this case, the items were taken from a study by Ruby [14] and the book “*Alimentação sem Carne*” (Eating without Meat) [2]; (c) time adopting the diet. The longer time of diet adoption may be associated with a better adaptation, positively influencing the QoL, as already demonstrated in a study that evaluated the QoL in celiac individuals, who also adopt a dietary pattern that involves the total exclusion of a food group [42]; (d) presence of close people who adopt the diet. Considering the influence of the social context on QoL, possibly having close people adopting the same eating patterns could be a positive contribution factor; (e) perception of the cost of the diet; and (f) places where food has a higher cost. As pointed out by Carson et al. [18], economic aspects associated with diet can negatively influence the QoL of individuals with limited financial resources.

**Figure 1:**
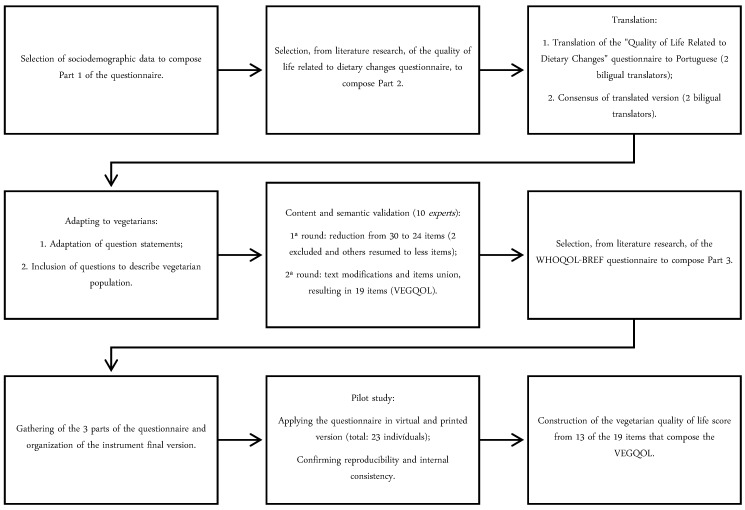
Flowchart of the vegetarian quality of life (QoL) questionnaire construction process.

The instrument structure was designed to be objective but still include all sufficient data to obtain the desired information. Figure 1 shows the flowchart for preparing the questionnaire. The full instrument is in Table 1.

#### 3.1.3. General Quality of Life

The third part of the instrument, in turn, focused on assessing the general QoL, without a direct correlation with the vegetarian diet. The goal was to generate data that could be compared with the QoL studies in other populations, unlike the evaluation made in part two, which was specifically focused on the effect of diet on the QoL. Since that instrument was produced specifically for vegetarians, comparative analysis with other population groups would be more complicated. Therefore, it was decided to apply the WHOQOL-BREF, an instrument that evaluates the QoL in a broad and general way.

### 3.2. VEGQOL Validation

#### 3.2.1. Content and Semantic Validation

The questionnaire was then submitted to content and semantic validation by the judges’ technique. Initially, the questionnaire consisted of 30 items (Figure 1). From the results obtained in the first round of judges’ evaluation, of the total of 30 items, 28 (93.3%) were considered APPROVED, according to the established parameter—“The item is considered approved (maintained in the instrument) when there is a minimum of 80% agreement between the judges for grades 4 and 5 (“partially agree with the item” and “fully agree with the item” respectively) [43].

Regarding the two items without consensus among judges, they were considered repetitive, confusing and/or not consistent with the questionnaire’s objective; therefore, they were removed from the instrument. All the evaluated items received comments, which we took into account for the questionnaire’s rewording. As many items were considered similar or repetitive, some questions were gathered and the number of items decreased from 30 to 24. We submitted the questionnaire second version to a second round to the judges evaluation, which included, in addition to the content evaluation, the semantic evaluation. At this stage, they evaluated the comprehension degree of each item.

From the second round’s results, five items were considered very similar to others and it was suggested to exclude those that were redundant. Of the 19 maintained items, all were considered APPROVED from the semantic point of view, according to the established parameter—“The item is considered approved (maintained in the instrument) when there is a minimum of 80% agreement between the judges for grades 4 and 5 (“I understood almost everything” and “I understood perfectly and had no doubts,” respectively) [43]. The VEGQOL modifications in the process of validation with all the excluded and modified items are presented in the Supplementary file (Table S1).

From the resulted 19 items after the judges’ analysis, six are about population characterization, as described above. The others (13 items) were used to compose the vegetarian QoL score. The vegetarian diet associated QoL questionnaire (VEGQOL) resulted in an instrument composed of 19 items (Table 1).

#### 3.2.2. Pilot Study and Questionnaire Validation

The printed version of the questionnaire was applied to a total of 28 individuals. Of these, 23 also answered the virtual version, sent three to four days after the application of the printed questionnaire. From the pilot study, it was possible to evaluate the acceptability of the questionnaire by missing items’ analysis. Regarding the physical questionnaire, of the 28 individuals, only one left one blank item out of a total of 90 items, which represents 1.1% of blank responses in 3.5% of the sample. In the virtual questionnaire, of the 23 respondents, three individuals left two blank items, representing 2.2% of blank responses in 13.0% of the sample. Considering the sum of items, 99.9% were answered in the physical questionnaire and 99.7% in the virtual questionnaire, indicating good acceptability [33].

The instrument’s reproducibility was verified item-by-item and also in the final score of the questionnaire. For the item-by-item analysis, the agreement between the two responses was measured using Cohen’s Kappa. For the Quality of Life score, the reproducibility of the questionnaire was verified using the Intraclass Correlation Coefficient (Table 2). From the comparison of the responses of the printed and virtual questionnaires of the 23 respondents to both, a good index of reproducibility of the instrument was observed. It indicates that the individuals, when they answered the questionnaire twice in non-consecutive days and without prior knowledge that it would be necessary to respond again in the same instrument, gave similar answers for both.

Internal consistency (reliability) of the VEGQOL (of the 13 items) was verified using the Cronbach Alpha’s measure, considering the physical instrument’s responses. The observed result was 0.708 (acceptable consistency) [36].

As shown in Table 2, items 11 and 13 presented a low reproducibility index. However, even after excluding them from the instrument, there was no significant change in internal consistency.

### 3.3. Score Construction

According to the obtained result from the judges’ technique, the 13 items that would compose the QoL score were defined. The pilot study was able to demonstrate the good internal consistency of items. For the score’s construction, a 1 to 5 scale was assigned for each of the five alternatives that would compose the score. The score of the items 5, 7, 11, 12, 13, 14 and 15 was inverted, that is, the first alternative corresponds to value 5, decreasing up to the last alternative, with value 1. The other items (6, 8, 16, 17, 18 and 19) have their value assigned according to the alternative number, also varying from 1 to 5. The total score corresponds to the sum of all items’ scores, ranging from 13 to 65, which corresponds to a 52 points’ difference. By convention, it was defined that the score would vary from 0 to 100, to facilitate results’ comprehension. Therefore, the final calculation of the score was done as follows:

$$\mathrm{Score}=\frac{\left(Sum\;of\;all\;items^{\prime }scores\right)-13}{52}\times 100.$$

### 3.4. Questionnaire Application (External Validation)

The questionnaire purposely asked, after every part, if the participant was willing to continue with the research or if they would like to end it at that point. This way, it would be possible to avoid data losses due to incomplete answers and it would still be possible to analyze each part separately. From the 5401 individuals that started to fill the questionnaire, a total of 5014 individuals replied up to the second part of the questionnaire (VEGQOL), being considered the final study sample. From these, 4375 (87.3%) also answered part 3, which corresponds to the WHOQOL-BREF. Sociodemographic characteristics of the population are presented in Table 3. Individuals who answer at least parts 1 and 2 were used to data analysis.

After the application of the questionnaire, internal consistency was verified using the Cronbach Alpha’s measure, this time using the final sample obtained. The observed result was 0.718 (acceptable consistency) [36]. Discriminant validity was obtained by the correlation between the results from the VEGQOL score and each domain of the WHOQOL, which showed a significant positive correlation for all domains, with similar results for each single domain (Supplementary file—Table S2).

### 3.5. Score Cut-off Points Definition

**Figure 2:**
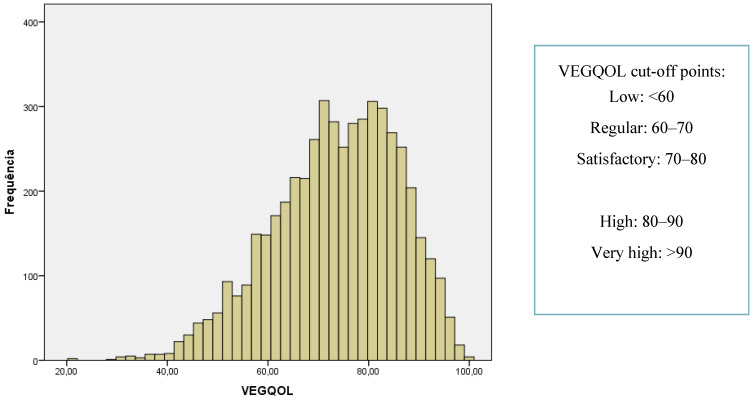
Definition of cut-off points and classification of VEGQOL scores.

It was possible to obtain a representative sample of the Brazilian vegetarian population, with a sufficient number of participants and proportional distribution in all the country regions, according to the estimated average of the real distribution on vegetarians in Brazil [44]. Therefore, since this sample represents the vegetarian population in Brazil, the results of the VEGQOL scores can be used to set cut-off points, based on the observed quartiles. It is possible, this way, to classify individuals’ scores into different categories, which helps the interpretation of data and comparison with future studies. The final result of this step can be found in Figure 2.

### 3.6. VEGQOL Results

The QoL scores results were analyzed according to the different characteristics of the studied population. A statistical difference was observed regarding gender, with female individuals showing a slightly higher score (females: 74.52 ± 12.43; males: 72.08 ± 12.48). Higher QoL was observed in older individuals (≥40 years old) compared to younger ones (<40 years old) (18–24 years: 71.67 ± 12.00; 24–29 years: 72.99 ± 12.14; 30–39 years: 74.47 ± 12.79; ≥40 years: 74.82 ± 12.72). Analysis by type of diet showed that the more restricted the diet, the higher the QoL was. Vegans showed better results (79.21 ± 10.66), followed by vegetarians (73.13 ± 11.58) and then, pesco-vegetarians (69.55 ± 12.50). Semi-vegetarians showed lower results (64.38 ± 12.84) among the participants. Moreover, the longer the diet was adopted, the better was the QoL. People who adopted the diet for less than one year reported lower QoL (70.21 ± 12.32) when compared to the ones who adopted the diet between one and five years (73.84 ± 12.05). Individuals adopting the diet for more than five years showed the highest scores (75.82 ± 12.71).

Regarding motivation for being vegetarian, the results showed that the ones who adopted it either for ethical of for health reasons had a better QoL (74.73 ± 12.21 and 73.05 ± 12.11, respectively). Individuals who were motivated by religion/beliefs or the reduction of environmental impact showed intermediate results (71.73 ± 12.66 and 72.06 ± 11.98, respectively). The ones who adopted a vegetarian diet due to aversion/intolerance to animal products (69.57 ± 14.26) or for other reasons (70.34 ± 13.88) were the ones who reported lower QoL. People who have relatives, partners, friends or colleagues who also adopt a vegetarian diet showed better QoL (74.57 ± 12.21) when compared to the ones who do not have close people adopting the diet (70.75 ± 12.89). All the results were statistically significant (*p* < 0.001) (Table 4).

When the cut-off points were considered for the mean analysis, vegans and vegetarians were classified as having satisfactory QoL, while pesco- and semi-vegetarians were classified as having regular QoL. Moreover, more than half (53.8%) of vegans had either good or very good QoL (score > 80), while the same result was observed in 31.4% of vegetarians, 22.8% of pesco-vegetarians and only 12.8% of semi-vegetarians. On the other hand, low QoL (score < 60) was observed in 39.7% of semi-vegetarians and in only 5.9% of vegans.

When motivation was analyzed by comparing the cut-off points, 38.8% of the individuals who adopted the diet for ethical reason had either good or very good QoL The ones who adopted the diet for personal health reasons of due to religion had similar results, with 32.8% and 31% of them classified as having good or very good QoL. The same classification was observed in 28.6% of the individuals who are vegetarians due to environmental concerns. The ones adopting the diet due to aversion/intolerance or other reasons had 24.7% and 28.3% of the individuals classified as having good or very good QoL, respectively.

## 4. Discussion

Evaluating quality of life (QoL) is a challenging process, since it relies on the individual’s subjective perception of their position in life, influenced by their culture and values and related to their goals, expectations, standards and concerns [45]. Therefore, it is essential to use accurate questionnaires to help quantify all the important parameters related to it. General QoL questionnaires, such as the WHOQOL [46] and the SF-36 [47], have already been developed and are very useful to evaluate and compare populations. However, when evaluating specific population groups, these tools might not consider important factors that can significantly influence the QoL, losing their power and sensibility. In fact, according to Ruano-Rodríguez et al. [48], generic QoL assessment tools may have limited applicability in the context of dietary changes. Therefore, developing specific questionnaires is necessary to evaluate QoL in different population groups correctly [49]. Dunn Galvin et al. [50] showed that it was possible to develop a specific questionnaire of food intolerance quality of life by adapting previously validated tools (in this case, a Food Allergy Quality of Life questionnaire) to the reality of the targeted study population. Having specific questionnaires to evaluate QoL can be very useful to help clinical decisions and implement public policies [50].

In order to develop a specific questionnaire for vegetarians (VEGQOL), another questionnaire, used by Delahanty et al. [28], was selected as a basis. In the original study, the questionnaire was aimed at individuals who were on a diet to control hypercholesterolemia. Although this type of diet, as well as a vegetarian diet, is also considered a restrictive food pattern, it was necessary to adapt the statements of some questions, as well as to include and exclude some items, based on other studies aimed at the vegetarian public for the adequacy of the instrument to this research.

The VEGQOL showed good results in the validation parameters—acceptability, internal consistency, reproducibility and discriminant validation. Moreover, it was developed by using the Delphi method for content and semantic validation [51], in which experts participated in the construction of the questionnaire, enriching its content and guaranteeing a more meticulous analysis and a better comprehension of its content.

Changing a diet pattern can bring either positive or negative effects on the QoL. Personal satisfaction and health improvement are examples of positive effects associated with diet changes. On the other hand, people who adopt a different diet pattern might face isolation and difficulties in maintaining social relationships, which may have a negative impact on their QoL [18]. For some specific diseases, for example, the treatment relies primarily on the adoption of a diet, which in most cases can be very restrictive and must be followed for the entire life, negatively impacting the individual’s life quality [20].

QoL related to dietary habits has already been evaluated in other contexts. A Mediterranean diet, which is not a strictly vegetarian diet but mainly a plant-based diet, has been positively correlated with QoL in Portuguese adolescents in a cross-sectional study [52]. Such correlation was also observed in another cross-sectional study with older Spanish overweight and obese individuals diagnosed with metabolic syndrome [53] and in patients with type 1 diabetes [54]. However, when health-related QoL was measured in two cohort studies with older males (>60 years old) in Spain, no clinically relevant association was found with Mediterranean diet adherence after a few years of follow-up. The cohorts were carried out 10 years apart from each other and diet adherence was measured by three different indexes, which assures more consistent results [55].

Many challenges can be faced when adopting a vegetarian diet, such as negative impacts on social relations and discrimination or social exclusion from non-vegetarians. In fact, social aspects are so relevant that the main reason for vegetarians to violate their diet is due to experiencing explicit pressure from friends, family, romantic partners and coworkers [56]. Other important factors such as difficulties in changing habits, enjoying the taste of meat, family or friends not being vegetarians, low access to vegetarian options when eating in restaurants and lack of knowledge regarding vegetarianism can also be considered barriers to adopt a vegetarian diet [21,57]. Moreover, the possible higher cost of vegetarian foods in some regions could also affect QoL. As pointed out by Carson et al. [18], economic aspects associated with diet can negatively influence the QoL of individuals with limited financial resources.

Therefore, it could be expected that people who chose to adopt a vegetarian diet might have a lower perception of QoL. On the other hand, vegetarianism can trigger positive feelings of peace and happiness, related to spiritual benefits, personal satisfaction, increased pleasure with the diet, environmental care, contribution to a more peaceful world and better QoL [21]. In a study conducted in the United States with workers of a company, volunteers adopted a vegan diet for 22 weeks and QoL was evaluated, as well as food acceptability and work productivity. Mental health and general satisfaction with the diet increased and they also saw improvements in general health, vitality and physical aptitude. However, participants reported having more difficulties finding options to eat out [21].

Our study showed that vegetarians in Brazil have a good QoL, according to our score. Sub-group analysis pointed out that the more restricted the diet, the higher the QoL. This result can be considered unexpected when compared to what can be observed from other studies with people who follow a restrictive diet, such as coeliac disease patients [58]. However, in this case, the diet restriction is imposed as a treatment for a condition, which can have negative impacts on the individual’s life. Vegetarianism, on the other hand, is mainly adopted due to a personal choice. Therefore, people who decide to adopt it might not feel as if they are being forced to abstain from eating animal products. Other results from our study also contribute to this idea, as it can be observed by the differences between motivations to adopt the diet. Individuals who adopt a vegetarian diet for ethical/moral reasons or for personal health had a higher average score when compared to the ones who adopted it because of aversion/intolerance. In this case, the first ones might feel like they are doing something positive to others (protecting animals) or to themselves (taking care of their health), which can bring more personal satisfaction and reflect in a higher QoL, opposing to the ones who simply adopt it because they do not like animal products (aversion) or due to intolerances. A qualitative study that investigated the relationship with food among vegan young female supports this theory. According to the study participants, becoming vegan for ethical reasons brought them a deep sense of belonging, as they started identifying themselves as part of the vegan community. Adopting a vegan lifestyle resulted in a positive impact on their relationships with themselves and with others [59].

A cross-sectional study conducted with runners in German-speaking countries also found good QoL levels in the participants. The WHOQOL-BREF was applied online to a total of 281 individuals (158 vegetarians, 123 omnivores). Results showed that both groups had high QoL, with no difference between vegetarians and omnivores. The authors concluded that runners have good QoL levels regardless of the type of diet and that a vegetarian diet is as good as an omnivore diet for runners regarding its effect in QoL [26].

Another factor that can influence QoL is an individual’s health state. Health problems, chronic diseases, excess weight and chronic pain are some factors that can affect an individual’s perception of life quality [45]. Vegetarianism has already been associated with better health outcomes and the prevention of chronic diseases such as obesity, type 2 diabetes, hypertension, ischemic heart disease and certain types of cancer [1]. It has already been described that vegetarian diets can help reduce body weight more than non-vegetarian interventions [60] and that vegetarians, especially vegans, have higher diet quality [61], which can positively influence their QoL perception [18]. A randomized control trial with diabetic patients evaluated the effect of a vegetarian diet on QoL and eating behaviors, compared to a standard diet used for type 2 diabetes treatment. Obesity and Weight-Loss Quality of Life – OWQOL and Weight-Related Symptoms—WRSM were the questionnaires used to evaluate the QoL. Both diets improved QoL but results with the vegetarian diet were better [20].

Our study also showed that vegetarians who adopt the diet for longer have higher QoL than the ones who adopt it for less time (less than one year). One explanation for this result can be the fact that it takes some time for individuals to adapt to a new diet pattern. Castilhos et at. [42] showed that coeliac patients who adopted the gluten-free diet for longer had a better QoL when compared to the ones who adopted it for less time, possibly due to a better adaptation to the restriction. We also found higher QoL scores in the ones who had close people also adopting the diet. Being surrounded by people with similar eating habits can make it easier to maintain them, especially when having meals together. According to Schmitt et al. [62], perceived discrimination has harmful effects on psychological well-being. Vegetarians tend to be stigmatized and the mere act of disclosing the choice of being vegetarian can cause anxiety and have a negative impact on social relationships [63]. Therefore, having close relationships with other vegetarians may reduce the possibility of suffering from discrimination.

Considering the substantial growth of vegetarians number in Brazil over the last years [10], campaigns that promote more information and provide options for individuals who adopt this type of diet are necessary. The Brazilian Vegetarian Society has taken important steps over the last years in order to spread actions related to vegetarianism. An example is the Meat-Free Monday (*Segunda sem Carne*) campaign, which is considered the biggest in the world and it is adopted in many governmental schools [64]. In partnership with state governments, 67 million meat-free meals were offered in schools in 2018 [64], increasing awareness about vegetarianism and contributing positively to the environment. In fact, even in non-vegetarians, better knowledge about the environmental impact of food production can influence consumers, leading them to choose more sustainable food options [6,65]. Moreover, due to the increase in vegetarianism popularity over the last years, industries, restaurants and big corporations have been following the trend, offering more vegetarian and vegan alternatives to fulfill the consumers’ demands [66]. These changes can bring a positive impact on vegetarians and vegans QoL, especially among those who adopt the diet due to aversion or intolerance to animal products, which are the ones who have lower QoL when compared to other vegetarians, according to our study.

A possible limitation of this study is the fact that the sample was composed mainly of female individuals (60.3%). However, due to the lack of official statistics regarding vegetarians in Brazil, it is not possible to know whether this number represents the real distribution of vegetarians or if other factors influenced it. As it has been already described, females tend to participate more in health surveys compared to male, since they are usually more concerned about health [43,67]. On the other hand, large studies such as the Epic-Oxford [68] and the Adventist-Health 2 Study [41] showed a similar trend, with 78% and 65% of the sample composed by females, respectively. Moreover, meat consumption is often associated with masculinity [69] and males are more resistant to going vegetarian compared to females [63]. Mullee et al. [70] showed that females are more likely to believe that meat consumption is bad for the environment and that vegetarianism is healthy and achievable. Therefore, we believe the gender distribution found in our study may reflect the general trend of a majority of vegetarians being females.

Using a convenience sample can also be considered a limitation of this study. However, if a random sampling was used instead, it would not be possible to achieve such a large sample, which allowed us to classify vegetarians in different categories to make sub-group analysis. Since our goal was to validate a new tool aimed at the vegetarian population, we purposely conducted online research with a convenience sample in order to be able to gather enough vegetarians for our study.

Our study sample was mainly represented by individuals living in urban areas (97.2%), which could influence the results. However, Brazil is mainly an urban country, with over 84% of the population located in cities [38]. The fact that this was an online conducted research might have facilitated reaching more individuals who live in urban areas and therefore have easier access to the internet. It is possible that more individuals in rural areas would adopt a vegetarian diet, mainly due to economic reasons and lower access to animal foods. Moreover, our sample participants had a high education level, which can also influence some of the QoL aspects. In fact, socioeconomic status has already been correlated with a higher QoL score [71]. On the other hand, it is possible that people with a higher education level are more prone to adopt a vegetarian diet, as it has already been previously described [68,72]. Future research should be conducted in order to evaluate the adoption of a vegetarian diet in rural areas and among people with lower education level and its potential influence on QoL.

To our knowledge, this is the first study to develop and validate a specific questionnaire to evaluate QoL in vegetarians, as well as to evaluate QoL among sub-groups of vegetarians on a nationwide basis. Much of the studies with this population group aim to evaluate specific dietary characteristics, such as nutritional deficiencies, nutrient intake levels and risks of chronic disease development, among other factors associated with health. However, there is a lack of data regarding the effect of vegetarianism on QoL. Studies with different population groups that adopt restrictive diets show that a narrower dietary pattern may negatively impact the QoL [20,42], contrasting with our results, in which vegetarians showed good QoL.

The final instrument produced by this study can be useful for evaluating specific features of QoL related to the vegetarian population, which was not possible in previous studies yet. Besides being useful for the Brazilian population, this tool can also be translated and culturally adapted to other countries, allowing more consistent and standardized data.

## 5. Conclusions

The VEGQOL showed good reproducibility, acceptability, internal consistency and discriminant validity, considered adequate to evaluate the QoL in vegetarians. Therefore, it can be considered a useful tool for future research in this area, in order to provide more accurate data related to a vegetarian diet and their possible effects in the individual’s QoL.

Based on our score, vegetarians showed good levels of QoL. According to subgroup analysis, QoL was directly related to the diet restriction level, being higher in the more restricted diet (vegan). Vegetarians who adopt the diet due to ethical/moral reasons or for personal health also showed higher QoL, which might be related to positive feelings triggered by doing something good for others (protecting animals) and for themselves (taking care of their health), respectively. Moreover, vegetarians who adopted the diet for longed and who had close people also adopting a vegetarian diet also had higher QoL. This result is related to the fact that social relations can affect QoL and it might take some time to adapt to a new diet pattern. In general, it is possible to say that, differently from other restrictive dietary patterns, vegetarianism does not seem to impact QoL negatively and the restriction might even be related to an improvement in QoL of individuals who adopt a vegetarian diet.

Understanding the effect of vegetarianism on QoL can support health professionals (doctors, dietitians, psychologists) in their conduct, leading to a better understanding of the context in which these individuals are inserted. Also, among all the aspects encompassed by the concept of QoL, to understand if and how vegetarianism impacts any of them are relevant so that institutions, public agencies and private entities can adopt tools and strategies aimed at assisting this public.

## Figures and Tables

**Table 1:** Vegetarian Quality of Life Questionnaire (VEGQOL) (Brazilian-Portuguese version and free translation to English).

Itens do Questionário (Brazilian-Portuguese)	Questionnaire Items (English)
1. Com relação ao padrão alimentar que você adota, como você o classifica? Vegano ou vegetariano estrito (não consome nenhum produto de origem animal) Vegetariano (não consome nenhum tipo de carne, mas consome ovos e/ou laticínios) Pescovegetariano (consome peixes/frutos do mar, mas não consome outros tipos de carne) Semivegetariano (adota uma dieta praticamente vegetariana, mas consome carnes menos de uma vez por semana)	1. Regarding the eating pattern you adopt, how do you classify it? Vegan or strict vegetarian (not consuming of any animal product) Vegetarian (not consuming of any type of meat but consuming of eggs and/or dairy products) Pesco-vegetarian (consuming fish/seafood but not consuming other types of meat) Semi-vegetarian (adopting a practically vegetarian diet but consuming meat less than once per week)
2. Há quanto tempo você adota a dieta vegetariana/vegana? Sempre adotei a dieta Há menos de 1 ano Entre 1 e 5 anos Há mais de 5 anos	2. For how long have you been adopting a vegetarian/vegan diet? I have Always adopted it For less than one year Between one and five years For more than five years
3. Qual foi a PRINCIPAL motivação que levou você a adotar uma dieta vegetariana? Ética/moral (com relação aos animais) Saúde pessoal Religião/crenças/espiritualidade Impacto ambiental Aversão, intolerância ou alergia a alimentos de origem animal Influência de outras pessoas (família, amigos, pessoas de convívio próximo) Outros(especifique):	3. What was the MAIN motivation for you to adopt a vegetarian diet? Ethical/moral reason (related to animals) Personal health Religion/beliefs/spirituality Environmental impact Aversion/intolerance or allergy to animal foods Influence of other people (family, friend of close people) Others(specify):
4. Pessoas próximas a você também adotam uma dieta vegetariana? (você pode assinalar mais de uma alternativa, se necessário). Sim, familiares Sim, cônjuge/parceiro(a) Sim, amigos e/ou colegas de trabalho Sim, outras pessoas próximas a mim Não.	4. Do people who are close to you also adopt a vegetarian diet? (you can mark more than one alternative, if necessary). Yes, Family members Yes, partner Yes, friends and/or work colleagues Yes, other close people No
5. Eu recebo críticas negativas pelo fato de seguir uma dieta vegetariana. * ^1^ Nunca recebo críticas negativas Raramente recebo críticas negativas Às vezes recebo críticas negativas Frequentemente recebo críticas negativas Sempre recebo críticas negativas	5. I get negative critics due to following a vegetarian diet. * ^1^ I never get negative critics I rarely get negative critics Sometimes I get negative critics I frequently get negative critics I always get negative critics
6. Eu recebo elogios pelo fato de seguir uma dieta vegetariana. * Nunca recebo elogios Raramente recebo elogios Às vezes recebo elogios Frequentemente recebo elogios Sempre recebo elogios	6. I get compliments for following a vegetarian diet. * I never get compliments I rarely get compliments Sometimes I get compliments I frequenly get compliments I always get compliments
7. Sinto-me constrangido ao comer de acordo com a dieta vegetariana, na presença de outras pessoas. * ^1^ Discordo totalmente Discordo parcialmente Não discordo nem concordo Concordo parcialmente Concordo totalmente	7. I feel embarrassed eating according to a vegetarian diet around other people. * ^1^ I totally disagree I partially disagree I neither agree nor disagree I partially agree I totally agree
8. Eu acho que o meu padrão alimentar é um bom exemplo para outras pessoas. * Discordo totalmente Discordo parcialmente Não concordo nem discordo Concordo parcialmente Concordo totalmente	8. I think my eating pattern sets a good example for other people. * I totally disagreeb. I partially disagreec. I neither agree nor disagreed. I partially agreee. I totally agree
9. Pensando no custo financeiro geral para a manutenção da dieta vegetariana em comparação a uma dieta não vegetariana, os alimentos que você consome são: Muito mais baratos Um pouco mais baratos O custo é mais ou menos o mesmo Um pouco mais caros Muito mais caros	9. Regarding the general financial cost for maintaining a vegetarian diet comparing to a non-vegetarian diet, foods that you consume are: Much cheaper A little cheaper The cost is about the same A little more expensive Much more expensive
10. Os alimentos que compõem a dieta vegetariana são mais caros em: Comércio (supermercados, mercados, feiras, lojas etc.) Alimentação fora de casa (restaurantes, bares, cafés etc.) Ambos Nenhum	10. The foods that compose a vegetarian diet are more expensive at: Commerce (supermarkets, markets, street markets, stores, etc.) Eating outside (restaurants, bars, coffee shops, etc.) Both None
11. Eu tenho dificuldade para adotar uma dieta vegetariana, devido ao seu custo mais elevado. * ^1^ Discordo totalmente Discordo parcialmente Não concordo nem discordo Concordo parcialmente Concordo totalmente	11. I have trouble adopting a vegetarian diet due to its higher cost. * ^1^ I totally disagree I partially disagree I neither agree nor disagree I partially agree I totally agree
12. A menor variedade de opções de alimentos no comércio (supermercado, mercados, feiras, lojas especializadas etc.) é uma das dificuldades que tenho para adotar uma dieta vegetariana. * ^1^ Discordo totalmente Discordo parcialmente Não concordo nem discordo Concordo parcialmente Concordo totalmente	12. The lower variety of food options commercially available (supermarkets, markets, street markets, specialized stores, etc.) is one of the difficulties I have in adopting a vegetarian diet. * ^1^ I totally disagree I partially disagree I neither agree nor disagree I partialy agree I totally agree
13. A menor variedade de opções de alimentação fora de casa (restaurantes, bares, cafés etc.) é uma das dificuldades que tenho para adotar uma dieta vegetariana. * ^1^ Discordo totalmente Discordo parcialmente Não concordo nem discordo Concordo parcialmente Concordo totalmente	13. The lower variety of food options when eating outside (restaurants, bars, coffee shops, etc.) is one of the difficulties I have in adopting a vegetarian diet. * ^1^ I totally disagree I partially disagree I neither agree nor disagree I partially agree I totally agree
14. Eu considero que refeições vegetarianas são mais difíceis de planejar que refeições não vegetarianas. * ^1^ Discordo totalmente Discordo parcialmente Não concordo nem discordo Concordo parcialmente Concordo totalmente	14. I consider that vegetarian meals are harder to plan than non-vegetarian meals. * ^1^ I totally disagree I partially disagree I neither agree nor disagree I partially agree I totally agree
15. Eu considero que refeições vegetarianas são mais difíceis de preparar que refeições não vegetarianas. * ^1^ Discordo totalmente Discordo parcialmente Não concordo nem discordo Concordo parcialmente Concordo totalmente	15. I consider that vegetarian meals are harder to prepare than non-vegetarian meals. * ^1^ I totally disagree I partially disagree I neither agree nor disagree I partially agree I totally agree
16. Quanto ao sabor dos alimentos que consumo na dieta vegetariana, eu me sinto: * Nada satisfeito Pouco satisfeito Razoavelmente satisfeito Muito satisfeito Extremamente satisfeito	16. Regarding the taste of the foods I eat on a vegetarian diet, I feel. * Not satisfied at all A little satisfied Reasonably satisfied Very satisfied Extremely satisfied
17. Eu sinto que, ao adotar uma dieta vegetariana, estou fazendo algo muito bom para o planeta (considerando meio-ambiente, animais, sociedade). * Discordo totalmente Discordo parcialmente Não discordo nem concordo Concordo parcialmente Concordo totalmente	17. I feel that, by adopting a vegetarian diet, I am doing something very good for the planet (considering the environment, animals, society). * I totally disagree I partially disagree I neither agree nor disagree I partially agree I totally agree
18. Eu sinto que, por adotar uma dieta vegetariana, sou mais feliz. * Discordo totalmente Discordo parcialmente Não discordo nem concordo Concordo parcialmente Concordo totalmente	18. I feel that, by adopting a vegetarian diet, I am happier. * I totally disagree I partially disagree I neither agree nor disagree I partially agree I totally agree
19. Eu sinto que, ao adotar uma dieta vegetariana, estou contribuindo positivamente para cuidar da minha saúde. * Discordo totalmente Discordo parcialmente Não discordo nem concordo Concordo parcialmente Concordo totalmente	19. I feel that, by adopting a vegetarian diet, I am positively contributing to take care of my health. * I totally disagree I partially disagree I neither agree nor disagree I partially agree I totally agree

* Vegetarian diet items associated with the quality of life scores. The others are items that characterize the studied population. ^1^ Items with an inverted score.

**Table 2:** VEGQOL reproducibility study.

	Agreements Measures	*p*-Value
Item 5	0.625 ^1^	<0.001
Item 6	0.643 ^1^	<0.001
Item 7	0.482 ^1^	0.001
Item 8	0.480 ^1^	0.001
Item 11	0.083 ^1^	0.567
Item 12	0.462 ^1^	<0.001
Item 13	0.097 ^1^	0.328
Item 14	0.361 ^1^	0.007
Item 15	0.362 ^1^	0.008
Item 16	0.730 ^1^	<0.001
Item 17	0.455 ^1^	0.001
Item 18	0.439 ^1^	0.007
Item 19	0.567 ^1^	<0.001
Indice QV	0.820 ^2^	<0.001

^1^: Cohen’s’Kappa. ^2^: Intraclass Correlation Coefficient (ICC).

**Table 3:** Sociodemographic characteristics of the studied sample.

Characteristic	Category	Respondents (*n* = 5014)	
		Number	Percentage
Gender	Male	1989	39.7%
	Female	3025	60.3%
Age	18–24	1412	28.2%
	25–29	845	16.9%
	30–39	954	19.0%
	40–49	1170	23.3%
	50–59	515	10.3%
	60 or more	118	2.4%
Housing location	Capital or metropolitan area	3377	67.4%
	Urban area (other cities)	1496	29.8%
	Rural area	141	2.8%
Average income	Less than two minimum wages ^a^	796	15.9%
	Between two and five minimum wages	1492	29.8%
	Between five and ten minimum wages	1344	26.8%
	Between ten and twenty minimum wages	794	15.8%
	Above twenty minimum wages	284	5.7%
	Not informed	304	6.1%
Educational level	No education	0	0%
	Elementary School, incomplete	4	0.1%
	Elementary School, complete	21	0.4%
	High School, incomplete	57	1.1%
	High School, complete	574	11.4%
	University level, incomplete	1295	25.8%
	University level, complete	3063	61.1%
BMI ^b^	<18.5 kg/m^2^	271	5.4%
	18.5 to 24.9 kg/m^2^	3247	64.8%
	>24.9 kg/m^2^	1445	28.8%
	Not informed	51	1.0%
Type of diet	Vegan	1559	31.1%
	Vegetarian	2391	47.7%
	Pesco-vegetarian	378	7.5%
	Semi-vegetarian	686	13.7%
Time adopting the diet	Less than 1 year	1253	25.0%
	Between 1 and 5 years	2182	43.5%
	More than 5 years	1579	31.5%
Main motivation	Ethic/moral	3032	60.5%
	Personal health	567	11.3%
	Religion/beliefs	242	4.8%
	Environmental impact	620	12.4%
	Aversion/intolerance	186	3.7%
	Others	367	7.3%
Close people also adopting the diet	Yes	3685	73.5%
	No	1329	26.5%

^a^ One minimal wage is equivalent to R$1045.00 or US$232.74 (in 2020). ^b^ Source: [39].

**Table 4:** Mean quality of life scores results and frequency of score categories of the studied population, by different characteristics, measured with the VEGQOL *n* = 5014.

Characteristic	Category	Low (<60)	Regular (60–70)	Satisfactory (70–80)	High (80–90)	Very high (>90)	VEGQOL Mean (SD) *	*p*
Gender	Male	17.9%	22.9%	29.2%	23.6%	6.3%	72.08 (12.48) ^A^	<0.001
	Female	14.5%	19.6%	27.3%	28.4%	10.2%	74.52 (12.43) ^B^	
Age	18–24	17.4%	24.4%	30.3%	23.2%	4.7%	71.67 (12.00) ^A^	
	25–29	15.1%	22.5%	30.2%	25.8%	6.4%	72.99 (12.14) ^A^	<0.001
	30–39	16.6%	16.6%	27.3%	29.1%	10.5%	74.47 (12.79) ^B^	
	40 or more	14.5%	19.9%	25.7%	28.1%	11.9%	74.82 (12.72) ^B^	
Type of diet	Vegan	5.9%	12.7%	27.6%	37.8%	16.0%	79.21 (10.66) ^A^	
	Vegetarian	14.3%	23.6%	30.7%	25.0%	6.4%	73.13 (11.58) ^B^	<0.001
	Pesco-vegetarian	23.5%	27.0%	26.7%	17.2%	5.6%	69.55 (12.50) ^C^	
	Semi-vegetarian	39.7%	27.0%	20.6%	11.2%	1.6%	64.38 (12.84) ^D^	
Time adopting the diet	Less than 1 year	21.9%	24.5%	29.9%	19.2%	4.5%	70.21 (12.32) ^A^	
	Between 1 and 5 years	14.6%	21.2%	28.6%	27.7%	7.9%	73.84 (12.05) ^B^	<0.001
	More than 5 years	12.7%	17.8%	25.7%	30.7%	13.1%	75.82 (12.71) ^C^	
Main motivation	Ethic/moral	13.5%	19.6%	28.1%	28.3%	10.5%	74.73 (12.21) ^A^	
	Personal health	16.8%	22.4%	28.0%	25.2%	7.6%	73.05 (12.11) ^AB^	
	Religion/beliefs	20.7%	24.4%	24.0%	24.8%	6.2%	71.73 (12.66) ^BC^	<0.001
	Environmental impact	17.1%	24.7%	29.7%	23.9%	4.7%	72.06 (11.98) ^BC^	
	Aversion/intolerance	23.7%	22.0%	29.6%	19.9%	4.8%	69.57 (14.26) ^C^	
	Others	24.8%	20.4%	26.4%	22.6%	5.7%	70.34 (13.88) ^C^	
Close people also adopting the diet	Yes	14.3%	19.5%	28.0%	28.7%	9.6%	74.57 (12.21) ^A^	<0.001
	No	20.2%	25.0%	28.2%	20.5%	6.2%	70.75 (12.89) ^B^	

* Categories with the same letters do not differ significantly.

## Supplementary Materials

The following are available online at https://www.mdpi.com/2072-6643/12/5/1406/s1, Table S1. Items that compose the Vegetarian Diet Associated Quality of Life questionnaire (VEGQOL): final result of the questionnaire after content and semantic validation by the judges’ technique. Original version in Brazilian-Portuguese; Table S2. Pearson correlation between VegQol and WHOQOL-BREF domains.
