# Supplementary material for: Brazilian Vegetarian Population—Influence of Type of Diet, Motivation and Sociodemographic Variables on Quality of Life Measured by Specific Tool (VEGQOL)
Source: Nutrients. 2020 May 14;12(5):1406. doi: 10.3390/nu12051406 (PMC7284834; doi:10.3390/nu12051406)
Supplement: Supplementary file 1 [file nutrients-12-01406-s001.pdf]

Supplementary file

Table S1. VEGQOL modifications in the content and semantic validation process by experts.

Items	Acceptance/Rejection/Modifications
Phase 1	
1. Com relação ao padrão alimentar que você adota, como você o classifica? / *Regarding the eating pattern you adopt, how do you classify it?*	Accepted
2. Há quanto tempo você adota a dieta vegetariana/vegana? / *For how long have you been adopting a vegetarian/vegan diet?*	Accepted
3. Qual foi a PRINCIPAL motivação que levou você a adotar uma dieta vegetariana? / *What was the MAIN motivation for you to adopt a vegetarian diet?*	Accepted
4. Você acha que a dieta vegetariana apresenta um padrão alimentar muito diferente de uma dieta não vegetariana (em termos de tipos de alimentos, variedade, opções), ou é basicamente a mesma coisa? / *Do you think that a vegetarian diet is a dietary pattern too diferente from a non-vegetarian diet (regarding types of foods, variety, options) or is it basically the same thing?*	This item was considered irrelevant and not very clear. It was removed.
5. Pessoas próximas a você também adotam uma dieta vegetariana? / *Do people who are close to you also adopt a vegetarian diet?*	Accepted
6. Os indivíduos com quem você normalmente socializa (em restaurantes, confraternizações, encontros, etc.) também adotam uma dieta vegetariana? / *Do people who you normally socialize with (at restaurants, parties, gatherings, etc.) also adopt a vegetarian diet?*	Very similar to question 5. Recommendation to remove it. It was removed.
7. Eu sinto dificuldade em comer com outras pessoas, pelo fato de adotar um padrão alimentar vegetariano. / *I have difficulties eating with other people due to adopting a vegetarian dietary pattern.*	Accepted
8. Eu percebo que a minha dieta vegetariana interfere negativamente nas minhas atividades sociais normais, com a família, amigos ou em grupo. / *I percieve that a vegetarian diet negatively interferes on my normal social activities, with Family, friend or groups.*	Accepted
9. Às vezes sou criticado por conta da alimentação que eu sigo. / *Sometimes I am criticized because of the diet I follow.*	Change the way it is written (not clear). Add an item with “compliments”
10. Às vezes me sinto estranho ao comer da minha maneira na presença de outras pessoas. / *Sometimes I feel weird by eating the way I do among other people.*	Change the way it is written. “Sometimes” makes the sentence ambiguous.
11. Eu tenho dificuldade em planejar e preparar refeições vegetarianas. / *I have difficulties in planning and preparing vegetarian meals.*	Split into two items. “Plan” and “prepare” are two different things.
12. Eu acho que os alimentos específicos para a dieta vegetariana custam mais que os outros alimentos. / *I think that specific foods for a vegetarian diet cost more than other foods.*	Change the writing.
13. Eu tenho dificuldade em pagar pelos alimentos específicos da dieta vegetariana, em mercados. / *I have difficulties in paying for specific vegetarian foods at groceries.*	Items 13 and 14 can be turned into one.
14. Eu tenho dificuldade em pagar pelos alimentos específicos da dieta vegetariana, em restaurantes. / *I have difficulties in paying for specific vegetarian foods at restaurants.*	Items 13 and 14 can be turned into one.
15. O custo dos produtos alimentícios para uma dieta vegetariana é algo que me afeta negativamente. / *The cost of foods for a vegetarian diet is something that negatively affects me.*	Change writing “negatively affects me” is very vague.
16. Eu tenho dificuldade em fazer as compras dos alimentos específicos para a dieta vegetariana. / *I have difficulties in purchasing specific foods for a vegetarian diet.*	Items 16, 17, and 18 can be turned into one. Change writing.
17. Eu tenho dificuldade para encontrar os alimentos que eu consumo nos mercados do meu bairro. / *I have difficulties in finding the foods that I eat in my neighborhood.*	Items 16, 17, and 18 can be turned into one. Change writing.
18. A falta de opções vegetarianas em mercados é algo que me afeta negativamente. / *The lack of vegetarian options at the groceries store is something that negatively affects me.*	Items 16, 17, and 18 can be turned into one. Change writing.
19. A falta de opções vegetarianas em restaurantes é algo que me afeta negativamente. / *The lack of vegetarian options in restaurants is something that negatively affects me.*	Items 19 and 20 can be turned into one. Change writing.

20. Eu tenho dificuldade para encontrar os alimentos que eu consumo em restaurantes. */ I have difficulties in finding the foods I eat in restaurants.*	Items 19 and 20 can be turned into one.
21. Eu gosto da maior parte dos alimentos que consumo. / *I like mosto f the foods I eat.*	Items 21 and 22 can be turned into one.
22. Estou muito satisfeito com o sabor dos alimentos que consumo. / *I am satisfied with the taste of the foods I eat.*	Items 21 and 22 can be turned into one.
23. Eu como os alimentos de que eu gosto sempre que eu quero. / *I eat foods that I like whenever I want to.*	Irrelevant, it is not correlated to the questionnaire objective. It was removed.
24. Alimentar-me da maneira que faço hoje me faz sentir bem. */ Eating the way I do today makes me feel good.*	Change the word "good" (too vague).
25. Eu acho que a maneira como me alimento hoje é um bom exemplo para outras pessoas. / *I think the way I eat today is a good example for other people.*	Accepted.
26. Comparado a antes da adoção da dieta vegetariana, eu percebo que hoje a minha saúde está melhor. / *Compared to the time before adopting a vegetarian diet, I perceive that my health is better today.*	Change writing (not clear)
27. Eu acho que a dieta vegetariana é muito boa para a minha saúde. / *I think that the vegetarian diet is very good for my health.*	Change writing. The word "good" is too vague.
28. Eu acho que estou fazendo algo bom para mim mesmo/a ao comer da maneira que eu como. / *I think that I am doing something good for myself by eating the way I do.*	Items 28 and 29 can be turned into one. Change writing.
29. Eu sinto que, ao adotar uma dieta vegetariana, estou fazendo o melhor possível para cuidar da minha saúde. */ I feel that, by adopting a vegetarian diet, I am doing the best I can to take care of my health.*	Items 28 and 29 can be turned into one. Change writing.
30. Em geral, eu cuido muito bem de mim mesmo. / *In general, I take good care of myself.*	Change writing. Too vague.
Phase 2	
1. Com relação ao padrão alimentar que você adota, como você o classifica? */ Regarding the eating pattern you adopt, how do you classify it?*	Accepted
2. Há quanto tempo você adota a dieta vegetariana/vegana? / *For how long have you been adopting a vegetarian/vegan diet?*	Accepted
3. Qual foi a PRINCIPAL motivação que levou você a adotar uma dieta vegetariana? / *What was the MAIN motivation for you to adopt a vegetarian diet?*	Accepted
4. Pessoas próximas a você também adotam uma dieta vegetariana? *Do people who are close to you also adopt a vegetarian diet?*	Accepted
5. Eu percebo que a minha dieta vegetariana interfere negativamente nas minhas atividades sociais normais, com a família, amigos ou em grupo. / *I percieve that a vegetarian diet negatively interferes on my normal social activities, with Family, friend or groups.*	Other factors could influence it. These aspects are already cited in other items. This item was considered irrelevant and removed.
6. Eu sinto dificuldade em comer com outras pessoas, pelo fato de adotar um padrão alimentar vegetariano. / *I have difficulties eating with other people due to adopting a vegetarian dietary pattern.*	Very similar to item 9. It was removed.
7. Eu recebo críticas negativas pelo fato de adotar uma dieta vegetariana. *I get negative critics due to following a vegetarian diet.*	Accepted
8. Eu recebo elogios pelo fato de adotar uma dieta vegetariana. / *I get compliments for following a vegetarian diet.*	Accepted
9. Sinto-me constrangido ao comer de acordo com a dieta vegetariana, na presença de outras pessoas. / *I feel embarrassed eating according to a vegetarian diet around other people*	Accepted
10. Eu considero que refeições vegetarianas são mais difíceis de planejar que refeições não vegetarianas. / *I consider that vegetarian meals are harder to plan than non-vegetarian meals.*	Accepted
11. Eu considero que refeições vegetarianas são mais difíceis de preparar que refeições não vegetarianas. / *I consider that vegetarian meals are harder to prepare than non-vegetarian meals.*	Accepted
12. Pensando no custo geral para manutenção de uma dieta vegetariana em comparação a uma dieta não vegetariana, os alimentos que você consome são: *Regarding the general financial cost for maintaining a vegetarian diet comparing to a non-vegetarian diet, foods that you consume are:*	Accepted

13. Os alimentos que compõe a dieta vegetariana são mais caros em: *The foods that compose a vegetarian diet are more expensive at:*	Accepted
14. Eu tenho dificuldade em adotar uma dieta vegetariana, devido ao seu custo mais elevado. / *I have trouble adopting a vegetarian diet due to its higher cost.*	Accepted
15. Uma dificuldade que enfrento por adotar uma dieta vegetariana é a menor variedade de opções de alimentos no comércio (supermercados, feiras, lojas etc.). / *A difficulty I face due to adopting a vegetarian diet is the lower variedy of food options in the commerce (supermarkets, markets, stores).*	Change writing.
16. Uma dificuldade que enfrento por adotar uma dieta vegetariana é a menor variedade de opções para comer fora de casa (em restaurantes, bares, cafés, etc.). / *A difficulty I face due to adopting a vegetarian diet is the lower variedy of food options when eating out (restaurants, pubs, coffee shops, etc.).*	Change writing.
17. Quanto ao sabor dos alimentos que consumo na dieta vegetariana, eu estou: / *Regarding the taste of the foods I eat on a vegetarian diet, I am:*	Change writing.
18. Eu me sinto mais feliz atualmente, depois da adoção da dieta vegetariana. / *I feel happier now, after adopting a vegetarian diet.*	Change writing.
19. Eu acho que a maneira como me alimento hoje é um bom exemplo para outras pessoas./ *I think that the way I eat today is a good example to other people.*	Change writing.
20. Minha saúde está melhor atualmente, depois da adoção da dieta vegetariana. / *My health is better now, after adopting a vegetarian diet.*	Items 20, 21, and 22 can be turned into one.
21. A dieta vegetariana contribui positivamente para a minha saúde. */ A vegetarian diet positively contributes to my health.*	Items 20, 21, and 22 can be turned into one.
22. Eu sinto que, ao adotar uma dieta vegetariana, estou fazendo algo bom para cuidar da minha saúde. */ I feel that, by adopting a vegetarian diet, I am doing something good to take care of my health.*	Items 20, 21, and 22 can be turned into one.
23. Eu geral, eu cuido muito bem de mim. / *In general, I take very good care of myself.*	Too vague and irrelevant. It was removed.
24. Eu sinto que, ao adotar uma dieta vegetariana, estou fazendo algo muito bom para o planeta (considerando meio-ambiente, animais, sociedade). / *I feel that, by adopting a vegetarian diet, I am doing something very good for the planet (considering the environment, animals, society).*	Accepted.
Phase 3	
Com relação ao padrão alimentar que você adota, como você o classifica? */ Regarding the eating pattern you adopt, how do you classify it?*	Accepted in this form
Há quanto tempo você adota a dieta vegetariana/vegana? / *For how long have you been adopting a vegetarian/vegan diet?*	Accepted in this form
Qual foi a PRINCIPAL motivação que levou você a adotar uma dieta vegetariana? / *What was the MAIN motivation for you to adopt a vegetarian diet?*	Accepted in this form
Pessoas próximas a você também adotam uma dieta vegetariana? *Do people who are close to you also adopt a vegetarian diet?*	Accepted in this form
Eu recebo críticas negativas pelo fato de adotar uma dieta vegetariana. *I get negative critics due to following a vegetarian diet.*	Accepted in this form
Eu recebo elogios pelo fato de adotar uma dieta vegetariana. / *I get compliments for following a vegetarian diet.*	Accepted in this form
Sinto-me constrangido ao comer de acordo com a dieta vegetariana, na presença de outras pessoas. / *I feel embarrassed eating according to a vegetarian diet around other people*	Accepted in this form
Eu considero que refeições vegetarianas são mais difíceis de planejar que refeições não vegetarianas. / *I consider that vegetarian meals are harder to plan than non-vegetarian meals.*	Accepted in this form
Eu considero que refeições vegetarianas são mais difíceis de preparar que refeições não vegetarianas. / *I consider that vegetarian meals are harder to prepare than non-vegetarian meals.*	Accepted in this form
Pensando no custo geral para manutenção de uma dieta vegetariana em comparação a uma dieta não vegetariana, os alimentos que você consome são: *Regarding the general*	Accepted in this form

financial cost for maintaining a vegetarian diet comparing to a non-vegetarian diet, foods that you consume are:	
Os alimentos que compõe a dieta vegetariana são mais caros em: *The foods that compose a vegetarian diet are more expensive at:*	Accepted in this form
Eu tenho dificuldade em adotar uma dieta vegetariana, devido ao seu custo mais elevado. / *I have trouble adopting a vegetarian diet due to its higher cost.*	Accepted in this form
A menor variedade de opções de alimentos no comércio (supermercado, mercados, feiras, lojas especializadas etc.) é uma das dificuldades que tenho para adotar uma dieta vegetariana. / *The lower variety of food options commercially available (supermarkets, markets, street markets, specialized stores, etc.) is one of the difficulties I have in adopting a vegetarian diet.*	Accepted in this form
A menor variedade de opções de alimentação fora de casa (restaurantes, bares, cafés etc.) é uma das dificuldades que tenho para adotar uma dieta vegetariana. / *The lower variety of food options when eating outside (restaurants, bars, coffee shops, etc.) is one of the difficulties I have in adopting a vegetarian diet.*	Accepted in this form
Quanto ao sabor dos alimentos que consumo na dieta vegetariana, eu me sinto: / *Regarding the taste of the foods I eat on a vegetarian diet, I feel:*	Accepted in this form
Eu sinto que, por adotar uma dieta vegetariana, sou mais feliz. / *I feel that, by adopting a vegetarian diet, I am happier.*	Accepted in this form
Eu acho que o meu padrão alimentar é um bom exemplo para outras pessoas. / *I think my eating pattern sets a good example for other people.*	Accepted in this form
Eu sinto que, ao adotar uma dieta vegetariana, estou fazendo algo muito bom para o planeta (considerando meio-ambiente, animais, sociedade). / *I feel that, by adopting a vegetarian diet, I am doing something very good for the planet (considering the environment, animals, society).*	Accepted in this form
Eu sinto que, ao adotar uma dieta vegetariana, estou contribuindo positivamente para cuidar da minha saúde. / *I feel that, by adopting a vegetarian diet, I am positively contributing to take care of my health.*	Accepted in this form

Table S2. Pearson correlation between VegQol and WHOQOL-BREF domains.

Correlations

		VegQol	Whoqol_D1	Whoqol_D2	Whoqol_D3	Whoqol_D4
VegQol	Pearson correlation	1	.386**	.392**	.302**	.330**
	Sig. (2 extremities)		.000	.000	,000	.000
	N	5014	4375	4375	4375	4375
Whoqol_D1	Pearson correlation	.386**	1	.691**	.467**	.491**
	Sig. (2 extremities)	.000		.000	,000	.000
	N	4375	4375	4375	4375	4375
Whoqol_D2	Pearson correlation	.392**	.691**	1	.593**	.531**
	Sig. (2 extremities)	.000	,000		.000	.000
	N	4375	4375	4375	4375	4375
Whoqol_D3	Pearson correlation	.302**	.467**	.593**	1	.440**
	Sig. (2 extremities)	.000	.000	.000		.000
	N	4375	4375	4375	4375	4375
Whoqol_D4	Pearson correlation	.330**	.491**	.531**	.440**	1
	Sig. (2 extremities)	.000	.000	.000	.000	
	N	4375	4375	4375	4375	4375

**. Correlation is significant at the 0.01 level (2-tailed).
